# When Does Rejection Trigger Aggression? A Test of the Multimotive Model

**DOI:** 10.3389/fpsyg.2021.660973

**Published:** 2021-06-25

**Authors:** Megan Stubbs-Richardson, H. Colleen Sinclair, Ben Porter, Jessica Weiss Utley

**Affiliations:** ^1^Social Science Research Center, Data Science for the Social Sciences Laboratory, Mississippi State University, Starkville, MS, United States; ^2^Social Science Research Center, Social Relations Collaborative, Mississippi State University, Starkville, MS, United States

**Keywords:** bullying, rejection, aggression, prosocial behavior, antisocial behavior, asocial behavior, self-harm, perceived groupness

## Abstract

Research has sought to identify the conditions under which rejection leads to retaliation. The Multimotive Model (MMM) proposes that there are three primary behavioral responses to rejection: prosocial (e.g., befriending others), asocial (e.g., withdrawal), and antisocial behavior (e.g., aggression toward others). In this study, we conducted the first full test of the MMM as well as expanded the model. Based on research linking aggression and “perceived groupness,” construal items were added assessing whether the rejection was perceived as extending beyond the individual to one's peers. We also included self-harm behavioral responses as this outcome was not sufficiently captured by existing antisocial or asocial operationalizations. This expanded model was then tested with two high school student samples (Ns of 231 and 374) who reported experiencing aggressive rejection (i.e., experienced physical, verbal, relational, or cyber aggression from peers). The MMM was compared to a saturated model separately in each of the two datasets using structural equation modeling. Results indicate that the saturated model provides a better fit for the data than the MMM across all models examined (all *p* < 0.001). In part, this is due to certain paths having different associations than hypothesized. For example, perceiving the rejection as carrying a higher cost was predicted to promote prosocial behavior, where instead it predicted asocial responses. Perceived groupness was the strongest predictor of antisocial responses. Self-harm outcomes were significantly and consistently associated with higher perceived costs across the models. These results and others will be discussed in the context of how we can better encourage prosocial and discourage antisocial and self-harm responses to social rejection, including bullying.

## Introduction

The Secret Service and Department of Education's joint report on school violence in the United States (Vossekuil, 2004) and related empirical research (e.g., Kupersmidt et al., 1995; Leary et al., 2006) support the finding that social rejection (e.g., bullying, cyberbullying, romantic rejection, ostracism) precedes aggressive behavior. Leary et al. (2003) asserted that a history of chronic or acute peer rejection underlies aggression in schools, including 87% of school shootings. However, most youths experience rejection but do not respond aggressively (Kass, 1999). Although much research has focused on the “rejection-aggression” link [see Hutchinson et al. (2008) for review], rejection can trigger anti-social, pro-social, asocial (Richman and Leary, 2009) or self-harm behaviors (Hinduja and Patchin, 2010). Accordingly, Blackhart et al. (2006) asserted that understanding when and why youth who experience rejection do vs. do not respond aggressively is a pressing question for rejection researchers.

To address this call, Richman and Leary (2009) proposed the Multimotive Model (MMM) which synthesized 40 years of research on the rejection-aggression link to identify moderating variables that could predict whether rejection triggers anti-, pro-, or asocial behavior. To our knowledge, this model is largely untested. In the present paper, we test the MMM (Richman and Leary, 2009) to identify when rejection leads to aggression as opposed to more prosocial or asocial responses. We also expanded the model to explore associations with self-harm related outcomes. Identifying the pathways from rejection experiences to retaliation and or self-harm could facilitate the identification of opportunities for intervention to prevent the escalation of violence in our schools.

### Background: Aggressive Rejection

Although several factors have been shown to increase aggression among adolescents, one of the key predictors of aggressive behavior is rejection (Leary et al., 2003). Rejection is a form of communication that conveys to the individual that there is something about him/her that is undesirable that warrants exclusion from social relationships/groups. Rejection can be expressed in multiple forms (e.g., physical or verbal aggression, bullying, shunning, or ostracism). Rejection can be active (where students are explicitly rejected or picked on directly by peers) or passive (where students feel invisible, left out). Whatever form it takes, the research is clear: rejection hurts (Eisenberger and Lieberman, 2004; Eisenberger, 2011; Landa et al., 2020). Chronic and acute social rejection have long-term negative psychological and physical consequences (Prinstein and La Greca, 2004; Modin et al., 2011; Gustafsson et al., 2012).

In the present study, we operationalized *aggressive rejection* as students self-identifying as having experienced physical, verbal, relational, or cyber aggression at the hands of one's peers. Physical aggression involves attempts to cause harm through hitting, shoving, or kicking others. Verbal aggression involves attempts to cause harm face to face by threatening another's self-concept, such as calling names. Relational aggression involves causing harm through gossip or exclusion from groups. Cyber aggression involves harming another through electronic means such as texting insulting messages or via sharing embarrassing social media posts. Bullied youth are thus included in our operationalization of rejected youth, as they are students who experience these forms of victimization repeatedly.

### School Safety and Responses to Aggressive Rejection

Schools are still one of the safest places for children in the United States (May, 2014). Anti-bullying and school violence reduction programs are effective at reducing victimization and violent behavior in schools (Musu-Gillette et al., 2018). Even with rates of victimization declining for youth, still American youth reported 749,400 victimizations (theft and non-fatal violent victimization) on school property and 601,300 incidents away from school property (Musu-Gillette et al., 2018). In a nationally representative study of school safety, one in five (21%) students in U.S. schools reported experiencing traditional bullying (e.g., physical, verbal, relational) while 8% reported experiencing cyber bullying (Musu-Gillette et al., 2018). In a national sample of youth (6th−10th grade), Wang et al. (2009) found the majority of youth to experience verbal bullying (54%), followed by relational (51%), physical (21%), and cyber bullying (14%).

The consequences of these victimization experiences impact multiple spheres of youth's lives, including their psychological, physical, and academic well-being (Esbensen and Carson, 2009; McDougall and Vaillancourt, 2015). And, perhaps not surprisingly, being the target of peer victimization can increase aggressive responding as youth engage in self-defense or retaliation (Frey et al., 2015; Stubbs-Richardson and May, 2020), contributing to a cycle of aggression in schools (Frey and Strong, 2018). Clearly, there is more work to be done to reduce aggression in schools and to improve school responses to bullying (Hinduja and Patchin, 2010, 2019).

Although rejection can lead to aggressive behavior (Leary et al., 2003), most individuals who experience rejection do not engage in aggressive behavior, instead responding with pro-social behavior (DeWall, 2010; DeWall and Richman, 2011; DeWall et al., 2011; Knowles, 2014) while others who experience rejection choose to withdraw (Schoch et al., 2015; Sommer and Bernieri, 2015). Further, some internalize—engaging in self-harm or suicide (Hinduja and Patchin, 2010)—rather than externalize by lashing out at others (Leary et al., 2003; Reijntjes et al., 2010). After all, lashing out when rejected is somewhat counterintuitive (DeWall and Richman, 2011; Reijntjes et al., 2011; Sinclair et al., 2011). When one experiences a social rejection, it presents a threat to the fundamental need to belong (Baumeister and Leary, 1995; DeWall and Richman, 2011). Aggressing in response to rejection does not increase the aggressor's likelihood of being accepted; in fact, aggression is more likely to lead to further rejection (Leary et al., 2006). Thus, it begs the question why an individual would choose to aggress at all?

Accordingly, a number of researchers have called for the need to address when and why rejection triggers aggression (Blackhart et al., 2006; DeWall and Richman, 2011; Sinclair et al., 2011). In response to this call, Richman and Leary (2009) proposed the MMM to explicate the rejection-aggression link. However, the model remains untested. We seek to remedy this matter in the present research.

### The Multimotive Model and the Rejection-Aggression Link

In the MMM, Richman and Leary (2009) suggested that individuals who encounter rejection are motivated to choose between three sets of behaviors. These options include: (1) prosocial behavior–seek acceptance due to heightened sense of desire for social connectedness; (2) antisocial behavior–lash out due to angry, aggressive urges related to self-defense or harming the rejection source; (3) asocial behavior–withdraw due to decreased sense of desire for social connectedness and to avoid future rejection and subsequent hurt feelings.

**Figure 1:**
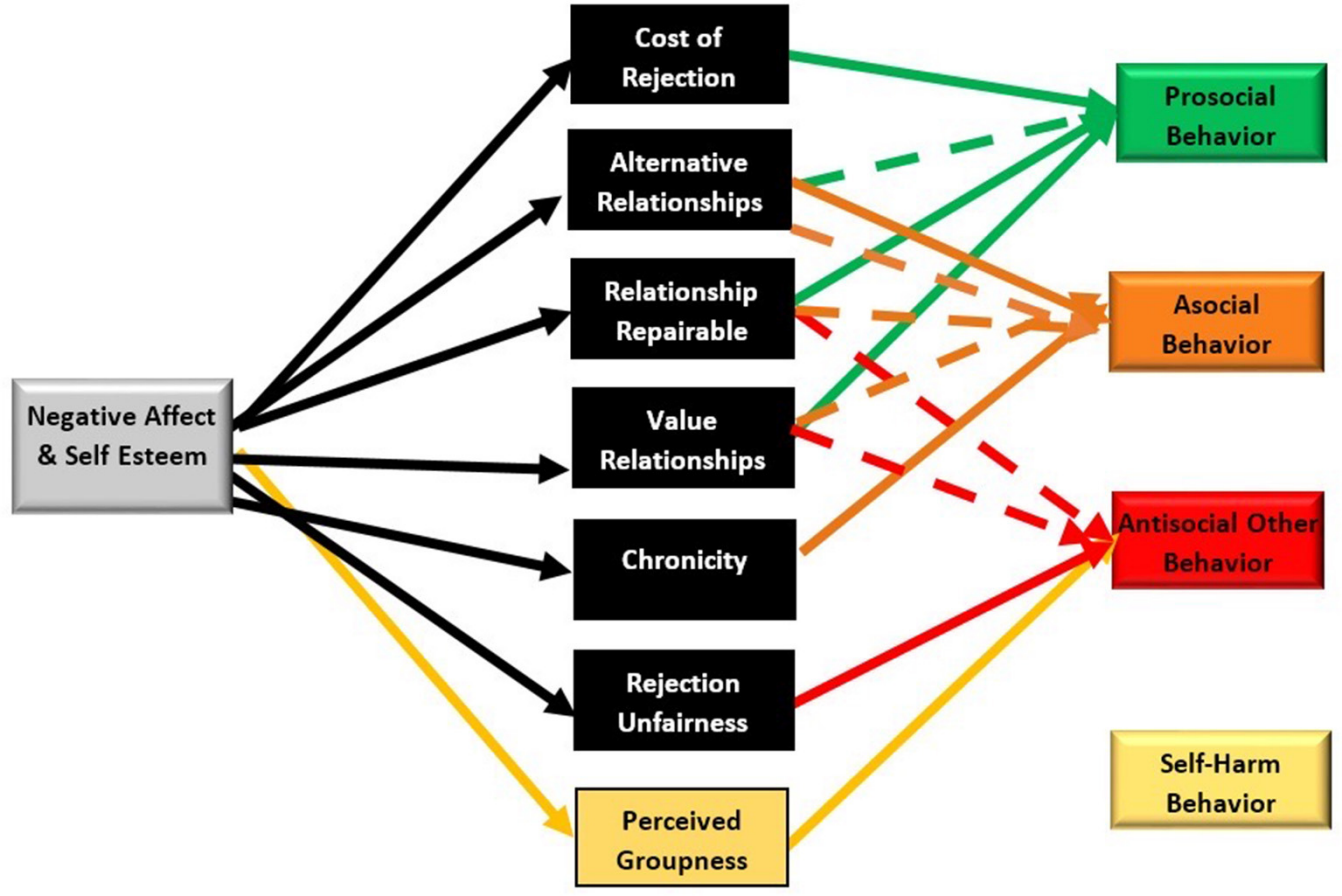
The modified multimotive model-predictions based on the multimotive model including anticipated groupness effect. Solid lines represents anticipated positive relationships. Dashed lines anticipated negative relaionships. Yellow lines and boxes were additions to the multimodel based on work on bullycide and perceivedgroupness effects.

According to the MMM, the behavioral response one chooses hinges on an individual's construal of the rejection experience. Construals include judgments about the perceived: (1) cost of rejection, (2) availability of alternative relationships, (3) likelihood of being able to repair the relationship, (4) relationship value, (5) chronicity, and (6) rejection unfairness (see Figure 1). For example, according to Richman and Leary (2009) the likelihood of an aggressive response is increased when rejection is perceived as unwarranted (e.g., unfair, insulting, unnecessarily rude, based on inaccurate information); one does not highly value relationships (does not fear what relationships s/he may lose from aggressing); or when one has little hope for relationship repair with the rejecter(s). Ultimately, the behavioral outcome chosen hinges on an individual's construals (i.e., their interpretation of the rejecting event). If this model holds true, potential interventions aimed at altering perceptions could facilitate reduction of aggressive retaliation.

### Rejection and Self-harm

When originally proposed, the MMM did not include self-harm as a possible outcome. Arguably, self-harm could be conceived as a sub-type of anti-social responding, just directed toward the self rather than others. Alternatively, it could be viewed as an extreme form of social withdrawal, particularly suicide, as ultimately one would be withdrawing completely from everything. Likely, it has some overlap with both constructs. However, as rejection and bullying both have been increasingly linked to self-harm and suicide (e.g., “bullycide;” Hinduja and Patchin, 2010, 2019), it was an important outcome to consider. Prior research conducted among a sample of 2,000 middle school students found traditional bullying victims (physical, verbal, relational) were 1.7 times and cyberbullying victims were 1.9 times more likely than non-victims to attempt suicide (Hinduja and Patchin, 2010). Youth who are both victims and bullies (i.e., “bully-victims”) were at the greatest risk for suicide (Hay and Meldrum, 2010).

### Rejection and Perceived Groupness

Rejection is a *social* phenomenon–it is a matter of how people relate. Aggression spurred by rejection does not occur within a vacuum. Thus, a model that focuses exclusively on individual impact may be missing context (i.e., group dynamics). Individuals can be targeted because of perceived group membership (Gaertner et al., 2008; Reijntjes et al., 2013; Utley et al., 2021). Likewise, an individual may choose to aggress against others in response to rejection by one because they perceive their rejecters as members of a group (Gaertner et al., 2008). Consequently, if the desired target is not available for victimization, displaced aggression–particularly aggression against those perceived as members of the “hated” group–occurs (Reijntjes et al., 2013).

Accordingly, we believe the MMM would benefit by taking “perceived groupness” (Gaertner et al., 2008) into consideration when trying to understand how rejection from one might trigger aggression against many. Gaertner et al. (2008) examined whether group membership of a rejecter was an important factor in experiencing rejection and found that participants were more likely to aggress against the rejecter when s/he was a member of a clearly defined group to which the participant did not belong [see also Schaafsma and Williams (2012)]. Participants generalized their aggression to other members of the group to which their rejectors belonged, even though those other group members had no direct involvement in the participant's exclusion. When the transgressing group is perceived as more cohesive (i.e., “they are all alike”), this displaced aggression is particularly satisfying to retaliatory aggressors (Sjöström and Gollwitzer, 2015). These findings overlap with a study of mass shooters' diaries and websites (Dutton et al., 2013). Researchers found evidence that mass shooters were obsessed with the perception that specific peer groups had unfairly wronged them (Dutton et al., 2013). For example, “Die Jock Die” was written on the backpacks of the Columbine shooters (Gaertner et al., 2008, p. 958) and Eric Harris was quoted as saying: “Isn't it fun to get the respect that we're going to deserve?” (Twenge and Campbell, 2003, p. 261).

Relatedly, those individuals who perceive they are rejected because of their own group membership are also more likely to engage in anti-social behaviors (Belmi et al., 2015). Lashing out is also more likely when an individual witnesses a member of their own group being targeted by others (Wesselmann et al., 2010; Coyne et al., 2011) because feeling empathy for the victim triggers defensive retaliation (Buffone and Poulin, 2014). In one study, targets of “connected victimization” [i.e., close connections with victimized peers; see also Peters et al. (2011)] were more likely to be disliked by their peers and were more likely to aggress than “isolated victims” (Zimmer-Gembeck et al., 2013). In another study, participants accompanied by co-targets who were excluded during a cyberball game were more aggressive toward rejecters than when sole targets, leading the researchers to conclude that when it comes to the impact of ostracism “there is no safety in numbers” (van Beest et al., 2012, p. 250). Based on this research we add a “perceived groupness” construal to capture the extent to which individuals felt their victimization was perpetrated by a group against their group.

### The Current Study

To our knowledge, the present research is the first test of the full MMM within a high school context. Past research on reactions to rejection has typically focused on only one type of behavioral outcome. Only presenting participants with one behavioral option, aggression (e.g., determine the level at which you wish to blast your rejecter with white noise), might artificially inflate the likelihood of that option being used. To better represent the choices that individuals have in the real world, the full spectrum of anti- to pro-social options needs to be available. In addition, our study has the added benefits of:

1. Addressing both direct and indirect victimization, both offline and online.
2. Adding self-harm outcome variables.
3. Considering the role of groupness construals.
4. Testing this model in a high school sample that is diverse, largely rural, and lower socioeconomic status.
5. Replicating the survey in two high school samples.

To test the modified theoretical model, we developed instruments specific to operationalizing the construals and behavioral responses. In Year 1, we ran an initial pilot study including these scales and modified them for the subsequent years. The pilot data can be found on the Open Science Framework (OSF, https://osf.io/7wyf3/). We then ran a Year 2 survey which we replicated in Year 3 with a sample recruited from our local high school via active consent procedures. All students were asked about their experiences with physical, verbal, relational, and cyber aggressive rejection in their school. Any student reporting an aggressive rejection experience was asked follow-up questions regarding how they construed the experience and then how they responded (prosocially, antisocially, asocially, or with self-harm). All codebooks are also available on the OSF. Structural equation modeling was then used to test the theoretical model. Hypotheses, for example predicted pathways specified by Richman and Leary, are in Figure 1 as well as included in **Table 3**. We used SEM to test the model's hypothesized links between construals and behavioral responses. We also anticipated a positive link between perceived groupness and aggressive behavior as indicated by research on group dynamics. As self-harm was not an outcome included in the Multimotive model originally, we had no hypotheses regarding the links between construals and self-harm and thus analyses were exploratory for this fourth type of behavioral response.

## Materials and Methods

### Demographics

We surveyed high school students about their experiences with physical, verbal, relational, and cyber aggression across three years (see Table 1 for operationalizations). Year 1 included pilot data and is not included in this research paper. Years 2 (*N* = 374) and 3 (*N* = 231), depicted in Table 2, consisted of participants from a rural southeastern public high school in the United States. In Year 2, 50% of participants identified as female, 39% as male, and 11% as other/refused. The mean age was 15.9 years (*SD* = 1.2). Racially/ethnically, 50.8% of participants identified as Black non-Hispanic, 25.9% as White non-Hispanic, 2.7% as Hispanic, and 11.2% as other race/ethnicity. Regarding class standing, 24% of participants were classified as seniors, 25% as juniors, 24% as sophomores, and 17% as freshmen.

**Table 1:** Definitions of types of bullying provided in survey of students.

Physical aggression	“Some students engage in physical aggression, such as hitting, kicking, and shoving other students. Physical aggression may also include any other attempts that have the potential to cause physical harm to another person.”
Verbal aggression	“Some students engage in verbal aggression, which includes face-to-face attempts to harm another person's self-concept. Examples include: calling others names or making fun of other.”
Relational aggression	“Some students engage in social aggression, such as spreading rumors about other students, purposely leaving people out of social groups or social events, turning people against each other, or giving the silent treatment. Social aggression may also include any other attempts to cause social harm.”
Cyber aggression	“Some students engage in cyber aggression, which includes virtual attempts to cause harm through social or digital media. Examples include: posting negative things about others online, posting unflattering pictures online, sending negative messages or threats via texts or the internet (e.g., Facebook), or sharing unflattering messages or pictures by text message or other social apps.”

**Table 2:** Demographic characteristics of the two datasets.

	**Year 2 dataset**	**Year 3 dataset**
	**(*N* = 374)**	**(*N* = 231)**
**Age**		
M (SD)	15.9 (1.2)	16.5 (1.5)
**Gender**		
Male	146 (39.0%)	90 (39.0%)
Female	187 (50.0%)	136 (58.9%)
Other/refused	41 (11.0%)	5 (2.2%)
**Race/ethnicity**		
Black non-hispanic	190 (50.8%)	134 (58.0%)
White non-hispanic	97 (25.9%)	57 (24.7%)
Hispanic	10 (2.7%)	14 (6.1%)
Other race/ethnicity	42 (11.2%)	24 (10.4%)
**Year in school**		
Freshman	65 (17.4%)	49 (21.2%)
Sophomore	89 (23.8%)	23 (10.0%)
Junior	95 (25.4%)	66 (28.9%)
Senior	90 (24.1%)	91 (39.4%)
**Most significant type of aggression**		
Physical aggression	92 (24.6%)	38 (16.5%)
Verbal aggression	124 (33.2%)	77 (33.3%)
Relational aggression	105 (28.1%)	76 (32.9%)
Cyber aggression	53 (14.2%)	40 (17.3%)

In year 3, 59% of participants identified as female, 39% as male, and 2% as other (see Table 2). The mean age was 16.5 years (*SD* = 1.5). Regarding race/ethnicity, 58% of participants identified as Black non-Hispanic, 24.7% as White non-Hispanic, 6% as Hispanic, and 10% as other race/ethnicity. Regarding class standing, 39% of participants were classified as seniors, 29% as juniors, 10% as sophomores, and 21% as freshmen.

### Materials

#### Emotional Responses

Participants completed a questionnaire asking about their experiences with physical, verbal, relational, and cyber aggressive rejection over the past 3 months. Participants were asked, “How often did someone from your school engage in physical/verbal/relational/cyber aggression toward you?” Participants responded to the question on a 6-point Likert-type scale, where 1 = *never* and 6 = *all of the time*. Participants were also given the option to decline a response. Students whose answers indicated they had experienced aggression from a classmate at least once were presented with questions assessing their emotional appraisal of the experience, such as whether it affected their self-esteem or resulted in any negative affect. In year 2, the constructs were combined into a single scale of 5 items which demonstrated good reliability (Y2: α = 0.93). In year 3, 6 items were included into the affect/self-esteem scale (Y3: α = 0.92). Note, for all variables, please see the Supplementary Table 1 for a list of items that were included or excluded across Years 2 and 3. Year 1 tests included pilot tests of newly created scales. In Year 2, as pilot testing showed some scales were still not strong enough, we added more items to strengthen the scales in Year 3. Ultimately, we added or removed items from scales to obtain the best measures possible for analysis. Thus, Year 3 scales were often shorter than Year 2 scales because, in order to reduce survey fatigue, only the strongest items from Year 2 were carried over to Year 3.

#### Construals

Participants then answered questions regarding their construal of the bullying they experienced.

Participants answered questions regarding their perceptions of the *chronicity* of their victimization for each type of victimization they experienced (e.g., “I feel like this type of aggression happens to me all of the time,” and “I feel like this aggression will continue no matter what I do”). In Year 2, three items were used to assess chronicity of victimization, and participants answered using a 7-point Likert-type scale, where 1 = *disagree strongly*, and 7 = *agree strongly*. Cronbach's alpha for reliability was 0.68. In year 3, the same three items were used to assess chronicity of victimization, using the same Likert scale. Cronbach's alpha for reliability was 0.83 in year 3.

Participants were asked questions about their perceived *relationship value*, assessing how much the rejection experience led them to value or devalue relationships in their life (e.g., “Because of this experience, I value the close relationships I have”). In Year 2, three items were used to assess relationship value, and participants answered using a 7-point Likert-type scale, where 1 = *disagree strongly*, and 7 = *agree strongly*. Cronbach's alpha for reliability was 0.80 for Year 2. In Year 3, the same three items were included, and participants answered using a 5-point Likert-type scale, where 0 = *not at all*, and 4 = *definitely/very much*. Cronbach's alpha for reliability was 0.86 in Year 3.

Participants were asked two to four items about *perceived fairness* of their victimization, assessing whether or not they perceived it to be unwarranted (e.g., “Do you think the actions this person/persons took toward you were mean?” and “Do you think the actions this person/persons took toward you were unfair?”). Participants responded using a 7-point Likert-type scale, where 0 = *completely fair* or *completely reasonable*, and 6 = *completely unfair* or *completely unreasonable* to a four-item scale in Year 2 and a two-item scale in Year 3. Cronbach's alpha for reliability was 0.86 in year 2, and 0.82 in year 3.

Participants were asked seven items about their perceived *costs of the rejection* in Year 2 and were asked 3 items in Year 3. These items assessed how participants perceived any negative effects that may have resulted from their victimization, including social costs (e.g., “How much did this experience have a negative impact on you?” and “How much did this experience cost you in a loss in reputation or status with friends/others?”). Participants responded to each item using a 5-point Likert-type scale, where 0 = *not at all*, and 4 = *definitely*. Cronbach's alpha for reliability in Year 2 was 0.91, and 0.87 in Year 3.

Participants were asked three items about their perceptions regarding *relational repair* in Years 2 and 3. These items assessed whether participants believed they may be able to repair the relationship with the person who victimized them, and have a positive relationship with them in the future (e.g., “To what extent do you have any interests in making the relationship you have with this person better?” and “To what extent do you feel you need to have a relationship with the person/persons who did this to you?”). Participants answered using a 5-point Likert-type scale, where 0 = *not at all*, and 4 = *definitely*. Cronbach's alpha for reliability was 0.91 in Year 2 and 0.92 in Year 3.

Participants were asked three items about their perceptions regarding *alternative relationships* in Years 2 and 3. These items assessed whether participants had other individuals they could turn to for social support (e.g., “To what extent do you have other people to whom you can turn to?” and “To what extent do you have other people who will support you?”). Participants responded to each item using a 5-point Likert-type scale, where 0 = *not at all*, and 4 = *definitely*. Cronbach's alpha for reliability was 0.95 in Year 2, and 0.95 in Year 3.

Participants were asked 2 items in Year 2 and 2 items in Year 3 about their perceptions of the extent to which *groupness* was involved in their reported victimization (e.g., “How typical is it for other members of your social group to be targeted by the same person(s) who harmed you?”). Participants responded to items on a 5-point Likert-type scale, where 0 = not at all and 4 = definitely. The scale showed acceptable reliability across the 2 years (Year 2 α = 0.84; Year 3 α = 0.81).

#### Behavioral Responses

Finally, participants were asked how they have responded to their reported physical, verbal, relational, and cyber aggression. In Years 2 and 3, participants answered four items to assess social withdrawal responses (e.g., “Trying to avoid situations where I have to be with other people”; α = 0.88 in years 2 and 3), three items to assess prosocial responses (e.g., “Trying to make new friends”; α = 0.84 in year 2; α = 0.83 in Year 3), and three items to assess antisocial responses in Year 2 (e.g., “Figuring out a way to get back at them”; α = 0.85 in year 2) and four items to assess antisocial responses in Year 3; α = 0.87 in Year 3). In Years 2 and 3, four items were used to assess self-harm responses (e.g., “Thinking about hurting myself”; α = 0.93 in year 2 and.92 in year 3).

### Procedures

For Years 2 and 3, consent and assent forms were prepared for each student enrolled in the school, labeled with the student's name, and distributed to classrooms by the researchers in two rounds. In order to participate, students had to sign the assent form, have a parent sign the consent form, and return the forms to school. Students were instructed to return the signed forms to the main office at school, where the research team would collect them. For returning signed consent and assent forms, students were allowed to choose a small incentive: either a metal water bottle, a USB drive, or a pair of earbuds. The research team used the signed consent and assent forms to compile a list of students, organized by grade, who would be called out of class to complete the survey over a 3-day period.

The research team set up laptop computers in the school auditorium (Y2) or in the cafeteria (Y3) to collect data. At least two seats were skipped between each laptop to facilitate confidentiality. Small groups of students were called out of class to complete the survey throughout the day. Each student's name was verified against the prepared list of students, given instructions for completing the survey, and stationed at a laptop computer. Members of the research team circulated the room during data collection to assist students who had questions, or if any technological issues arose.

Once students completed the survey, they returned to the member of the research team who checked them into the survey. Students were given the opportunity to choose a $10 gift card from Amazon, Apple, or Wal-Mart as compensation for their participation. Students signed a voucher acknowledging they received their gift card and were given a hall pass to return to class.

### Analytical Approach

The current manuscript tested the MMM separately in these two samples by comparing the MMM with a mostly saturated model (i.e., a model in which all paths between construals and outcomes were freely estimated). Because these two models are nested, a likelihood ratio test can compare the saturated and MMM. This is a direct test of the MMM with significant results indicating that the MMM does not fit the data. All residual covariances between construals were freely estimated as were all residual covariances between behavioral responses. In the MMM, all paths with a specified valence (i.e., positive or negative) were restricted to correspond to this valence. Given that groupness was not a component of the original MMM, associations including groupness were estimated without any constraint on the path.

Due to issues regarding psychometric fit of scales, two sets of analyses were run in each dataset with the sets of analyses differing by construct measurement with one derived using CFA and the other including all available items. However, the results were similar so only the results of the constructs made using CFA are reported (additional set of results available in Supplementary Table 2).

Initial analyses used CFA to ensure adequate measurement for each construct. For a construct to be considered a sufficient measure, all factor loadings must have been ≥0.7 (indicating ~50% of variance in the item was explained by the latent factor) as well as one of the following indicators of fit: RMSEA below 0.05; RMSEA below 0.08 with CFI and TLI >0.95; or a non-significant chi-square measure of fit. If the measurement model did not fit, items with a loading <0.6 were removed one at a time. If the model still failed to meet criteria, modification indices were used to determine whether residual covariances can improve fit. Residual covariances were added to the model one at a time until the above criteria were met or the modification index for adding a residual covariance was <4. If the measurement model still did not fit, the items with a loading >0.7 were retained. If only two items remained, the loadings of both were restricted to be equal to ensure constructs were locally identified.

Given the interest in self-harm reduction, an additional set of analyses were calculated in which self-harm behaviors were included in the saturated models as an additional behavioral outcome. More information can be found on the analysis plan and model parameters can be found on the Open Science Framework (https://osf.io/7wyf3/).

## Results

### Modified Analysis Measurement Models

**Figure 2:**
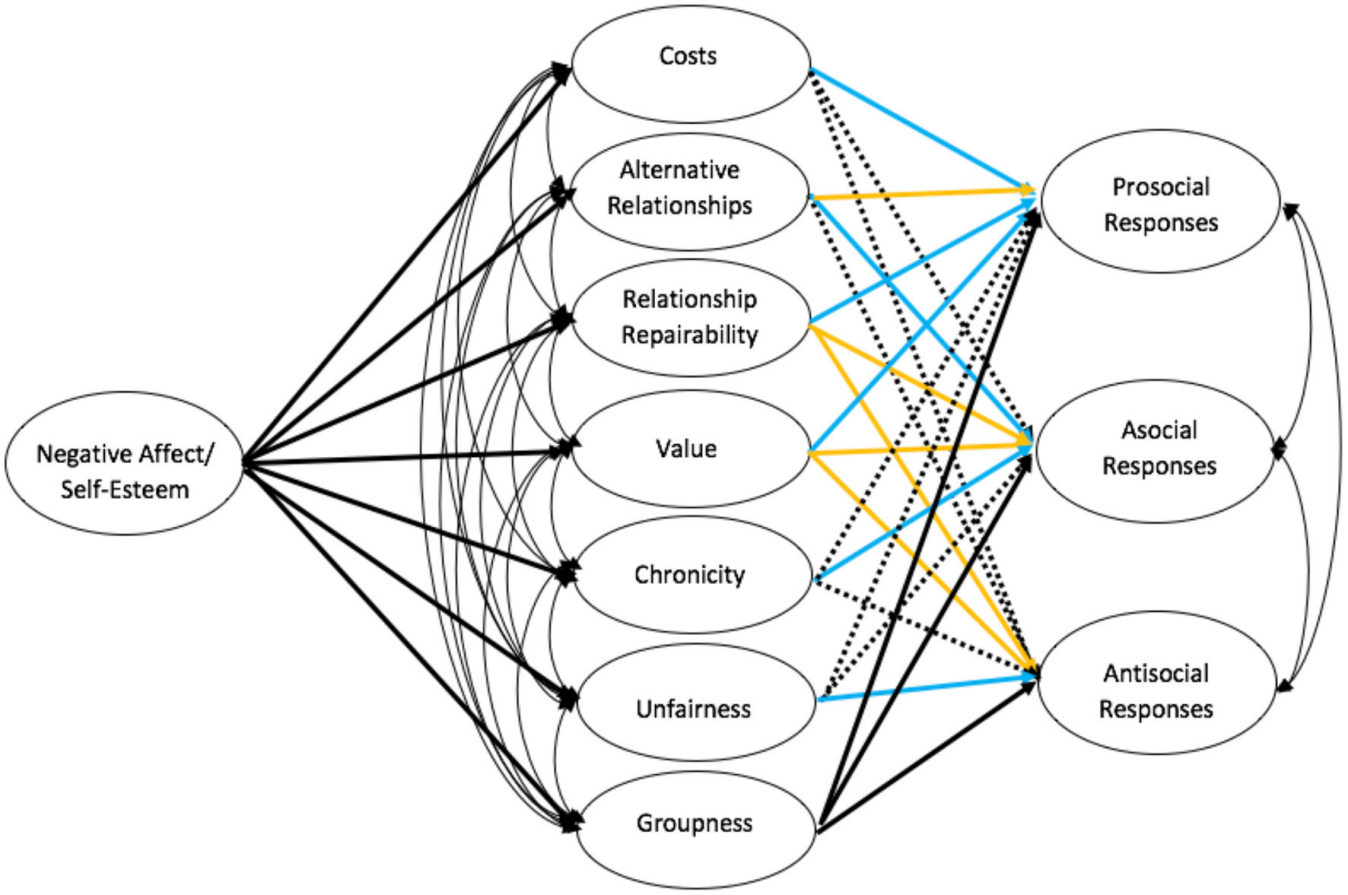
Structural paths and covariances between latent variables are shown in the model, but not measurement paths. Paths estimated in both the Multimotive Model and saturated model are solid. Blue lines indicate a path that was restricted to be positive in the Multimotive Model and orange lines indicate a path that was restricted to be negative. Dotted lines indicate a path was only estimated in the saturated model.

The items included in each latent variable for each dataset are listed in Supplementary Table 2. The difference between measures was generally due to items that were close to the predetermined threshold and were over the threshold in one dataset but not others (e.g., cost of rejection). The latent variables were exactly or almost exactly identical across the two datasets indicating the latent measures capture the same core concept. Structural paths and covariances are depicted in Figure 2.

### Modified Analyses

The analyses indicated that the mostly saturated model fit the data better than the MMM in Year 2 [χ^2^(7) = 41.3, *p* < 0.001] and [Year 3: χ^2^(7) = 51.3, *p* < 0.001]. The saturated model had had good fit in year 2 (RMSEA = 0.050, CFI = 0.94, TLI = 0.93, SRMR = 0.06) and Year 3 (RMSEA = 0.049, CFI = 0.94, TLI = 0.94, SRMR = 0.05). Of note, despite fitting more poorly than the saturated models, the MMM had adequate measures of fit in Year 2 (RMSEA = 0.051, CFI = 0.94, TLI = 0.93, SRMR = 0.07) and Year 3 (RMSEA = 0.052, CFI = 0.94, TLI = 0.93, SRMR = 0.07).

Negative affect/self-esteem was related to all construals (|*B*| > 0.25, *p* < 0.001) for all associations except for the association between alternative relationships regressed on negative affect/self-esteem in year 2 (*B* = 0.08, *p* = 0.14). The saturated model indicated several paths that were in the opposite direction than predicted by the MMM (see Table 3). Specifically, predicting asocial responses, alternative relationships and relationship value were in the opposite direction than predicted. Predicting antisocial responses, relationship repairability and relationship value (Year 2 only) were in the opposite direction than predicted. Groupness was not significantly associated with prosocial or asocial responses, but was the strongest predictor of antisocial responses (β'*s* = 0.23 and 0.35).

**Table 3:** Standardized structural path loadings from modified analyses, all paths estimated.

		**Predicted direction**	**Year 2**	**Year 3**
Prosocial responses	Cost	+	0.12	0.04
	Alternative Relationships	–	0.14^*^	0.11
	Relationship repairability	+	0.10	0.18^*^
	Value	+	0.24^***^	0.27^**^
	Chronicity	0	0.22^*^	0.12
	Unfairness	0	0.04	0.07
	Groupness	±	−0.04	0.04
Asocial responses	Cost	0	0.29^***^	0.53^***^
	Alternative relationships	+	−0.06	−0.09
	Relationship repairability	–	−0.02	−0.01
	Value	–	0.13^*^	0.07
	Chronicity	+	0.17^*^	0.22^*^
	Unfairness	0	−0.01	−0.03
	Groupness	±	0.05	−0.14
antisocial responses	Cost	0	0.18^*^	0.15
	Alternative relationships	0	−0.11	−0.08
	Relationship repairability	–	0.06	0.09
	Value	–	0.01	−0.02
	Chronicity	0	0.05	−0.04
	Unfairness	+	0.02	0.04
	Groupness	±	0.23^*^	0.35^**^

* *p < 0.05*;

** *p < 0.01*;

*** *p < 0.001*.

### Associations With Self-harm

In the Year 2 model (RMSEA = 0.050, CFI = 0.94, TLI = 0.93, SRMR = 0.06), self-harm was associated with cost (β = 0.57, *p* < 0.001) and unfairness (β = 0.10, *p* = 0.048). In the Year 3 model (RMSEA = 0.049, CFI = 0.94, TLI = 0.93, SRMR = 0.05), self-harm was associated with cost (β = 0.56, *p* < 0.001), and chronicity (β = 0.20, *p* = 0.02).

## Discussion

A better understanding of why youth respond to aggressive rejection can improve school responses to peer aggression, including bullying prevention programs (Frey et al., 2015). One way to begin to decrease aggressive responses connected to rejection is to understand which factors make youth more likely to respond aggressively compared to prosocial responding. Thus, this would allow for the development of interventions that discourage the former and encourage the latter. In the current study, we tested a novel theoretical model that hypothesized relations between certain perceptual factors and antisocial (retaliatory) behavior compared to prosocial (befriending others), and asocial (avoiding social events or people) responses to rejection. Although only a handful of the variables identified by the model proved useful in the predicted directions, we did find some significant relationships between factors included in the MMM and, specifically, for prosocial responding. Further, our amendment to the model wherein we included means to assess the perceived groupness of the rejection proved useful in predicting antisocial responses. Lastly, our addition of self-harm as a fourth type of behavioral response to aggression provides some groundwork for future studies examining this outcome.

### Key Findings

The self-esteem and negative affect predictors were significantly associated with all construal's in the model, except for alternative relationship in Year 2. However, as noted, the MMM did not play out according to many of its predicted pathways for Year 2 or 3 data, and few hypothesized associations were significant. None of the hypothesized associations in the MMM were significant predictors of aggression.

Speculation about failure to reach significant levels should be made with caution. The absence of a finding doesn't mean there were not existing relationships, rather just that they were not found using the existing sample, method, and instruments. The work on the rejection-aggression link, however, typically only examines one outcome (e.g., antisocial behavior, prosocial behavior, *or* self-harm behavior) where participants are not given the full spectrum of behavioral responses available to them outside of a laboratory setting. As such, studies upon which the theory was based may be suffering from a sort of mono-operational bias, though not necessarily due to the use of a single measurement but rather due to the examination of a singular outcome (even if measured multiple ways, e.g., aggressive thoughts and aggressive behavior). If only given a hammer, participants see everything as a nail, so to speak. As such, the likelihood of aggressive responding might be inflated in past studies, but as participants were not given other options, we do not know if they would have chosen to reach out instead of lash out. A model that predicts pathways between rejection and different outcomes might be better grounded in research that allows for multiple behavioral responses - not just to use a hammer or not use a hammer–in their methods.

As we provide the first test of the full model, however, it remains to be seen if different measures, methods, or samples might yield different results. For example, prior studies on which the MMM was based also consisted primarily of participants who were primarily white, educated, industrialized, rich, and democratic. Meanwhile, our study applies MMM to explain youth responses to aggressive rejection in a low income, racially diverse, and rural, Southeastern high school context. As such, we recommend future tests of this model be applied to different populations of study (e.g., adults) that also address an array of rejection types (e.g., romantic rejection, workplace rejection, discrimination) and employs an experimental design.

Nevertheless, there were some significant associations for each of the four outcomes: prosocial, asocial, antisocial, and self-harm that can inform theory and practice for anti-bullying interventions. For example, the results from the saturated models suggested that reducing victims' perceptions of the costs of aggressive rejection may reduce self-harm and asocial behavior. Further, addressing the group dynamics—such as whether individuals are targeted because of their group identity—could further help reduce aggressive responses. We discuss these and other significant pathways and then we discuss theory and policy implications for those associations.

Relational repair (i.e., perceptions of the likelihood that one could restore a relationship with the rejecter) and valuing relationships were two consistent significant predictors of prosocial responding across Years 2 and 3. Alternative relationships (i.e., having other relationships, especially supportive relationships) was also a significant predictor of prosocial responding in Year 2. In the modified analyses, relational value also held up as a significant factor in Years 2 and 3. These findings point to the possibility of teaching youth the importance of relationships and could help motivate prosocial over antisocial responses when rejected. For example, anti-bullying programs based on Social-Emotional Learning (SEL) provide evidenced based approaches to helping youth build skills in self-awareness, self-management, social awareness, relationship skills, and responsible decision-making. Further, SEL based programs have success in reducing problem behaviors in school, such as bullying. These programs are effective because they give youth the skillsets that they need to better engage in conflict resolution and relationship repair when problems are presented (Li et al., 2011; Guo et al., 2015; Oberle et al., 2016; Stalker et al., 2018).

Costs of the rejection (e.g., perceiving a loss in status/friendship/reputation) was the only significant factor that upheld across Years 2–3 for asocial responding. The greater the costs, the more likely students were to retreat. Relationship value and perceived chronicity were also significant in one of the 2 years. This pattern persisted in the modified analyses with the exception of chronicity being significant across both samples. Thus chronic, costly rejection experiences appear to promote social withdrawal. To re-engage youth, measures could be put in place to ameliorate perceptions of the costs associated with the experience, and to implement interventions that reduce aggressive rejection in the schools particularly for youth who are frequent targets.

When it comes to antisocial responding, the only significant predictor was perceived groupness (i.e., perceiving the rejection as extending beyond just a rejection of the individual to also being a rejection of their friends or social identity) and cost in 1 year of the modified analyses. Perceived groupness was not originally included in the MMM but is one we felt was important to add based on a line of research finding this factor to be associated with aggression (Twenge and Campbell, 2003; Gaertner et al., 2008). The importance of this variable could be indicating the presence of co-victimization (Schaafsma and Williams, 2012; Sjöström and Gollwitzer, 2015), such that youth are accurately perceiving that those they care about are also being rejected and victimized. Alternatively, it could be that youth are perceiving that they belong to a group marginalized by school culture. Either way, intergroup conflict theories, such as social identity theory, could thus be useful to integrate into more research on aggressive rejection, including bullying to highlight potential paths for intervention. Considerable work has been conducted on how to improve intergroup relations in the presence of conflict which could inform interventions.

The most consistently significant factor linked to self-harm was costs. Self-harm was also associated with relational repair, unfairness, and groupness, although inconsistently across the 2 years of data. In the latent model, costs and unfairness were significant while in the Year 3 latent and manifest models, costs and chronicity became significant. It appears then that self-harm bears more similarity to asocial responding than antisocial responding at least in terms of the factors to which it is connected. Self-harm, including risky behavior and suicidal ideation, may be on the extreme end of a continuum of asocial responses where perceived cost of the rejection is the strongest link.

### Prior Research and Novelty

This study offers the first test of the MMM (Richman and Leary, 2009) among a sample of students in a Southeastern high school. The MMM set out to explain when rejection leads to antisocial, asocial, and prosocial behavior. While many of the hypothesized paths in the MMM were not supported by the current study, we identified several other characteristics that may be incorporated into future interventions.

Additionally, we extended the model to also include a self-harm outcome, as many studies find a link between bullying and self-harm (Hay and Meldrum, 2010; Hinduja and Patchin, 2010). Our study also revealed the importance of perceived costs in terms of increasing the likelihood of self-harm. Thus, affecting either perceptions of costs or instrumentally reducing costs (e.g., compensating students for lost material costs where applicable) could help address both social withdrawal and prevent self-harm.

Further, our addition of perceived groupness proved to be a significant predictor to include in the model, particularly since it was the only variable significantly linked to aggression. It is noteworthy that it is one's group identity, as opposed to the rejecter's perceived groupness, that was associated with antisocial responding. Meaning, it was the extent to which individuals felt they were being targeted as part of a group rather than they were being targeted by a group that led to retaliation. Perhaps then youth are retaliating out of the perception that they are protecting their peer group rather than simply engaged in self-defense (Stubbs-Richardson and May, 2020). Defending others has more noble associations than personal revenge.

### Shortcomings and Limitations

Some of the study limitations include that neither Years 2 nor 3 provide large samples. However, both studies included a full consent procedure where both parents and students had to consent and assent for student participation.

The generalizability of the sample is limited given that this study was conducted in one Southeastern high school. Still many studies on rejection and bullying do not include diverse samples. Our study included 51 to 58% of students who identified as African American across Years 2 and 3. African American samples are often overlooked (Peskin et al., 2006) in studies on bullying in high schools and in studies on the rejection-aggression link. Another limitation is that our data makes use of self-report survey methodology which required the development of all new scales to test the MMM. Year 1 allowed us to pilot and improve some of the measures included in the model prior to testing the data in Years 2 and 3, but some measures could likely be improved further. Nevertheless, we believe the replication of findings uncovered in Years 2 through 3 helps to reduce some of the limitations found in creating new scales and using self-report methodology, and it strengthens the findings overall. Finally, the reports of victimization in the current study are reflective of the actual experiences that students have providing increased external reliability; however, this also meant that reported experiences vary widely across the sample.

### Theoretical Implications

We found the MMM not to be a good fit in terms of explaining antisocial and asocial responding; however, it does a better job explaining prosocial behavior. Although one factor that explained increased prosocial behavior—alternative relationships—was proposed to explain an increase in asocial behavior, not prosocial behavior. We believe future research should use the model to test a variety of types of rejection (e.g., romantic) across varying age samples to see if different results are met with the MMM. Further variations in the operationalization of different MMM variables could be employed.

In terms of using the MMM to explain responses to aggressive rejection, we also believe testing this model in other samples should be conducted to ensure our findings are not specific to a Southeastern rural high school context. However, based on some of our findings, perceived groupness should be included in future tests of the model to explain the likelihood of antisocial behavior. What proved important for the perceived groupness variable was how much youth felt like they—and notably their friends—were being targeted because of their group identity. Follow-up studies should continue to include the perceived groupness of the rejecter given experimental studies have shown this factor to matter (Gaertner et al., 2008). Further, the inclusion of assessment of both victim and perpetrator group identity variables would be consistent with classifying aggressive rejection in schools as an intergroup conflict.

### Policy Implications

Overall, declines in school aggression and bullying over time may in part be due to successful bullying prevention programs in schools. From 2015 to 2016, 76% of schools offered training for school personnel on the types of bullying, including physical, relational, and verbal (Musu-Gillette et al., 2018). More can be done.

Our research can inform prevention programs in a number of ways. Specifically, our findings would suggest that there is a need to reduce the perception of perceived costs (loss in reputation or status), perception that one's peer group or friends are being attacked (perceived groupness), and improve school relationships by teaching students conflict resolution skills which have been shown to be an effective component of prior anti-bullying prevention programs (Frey et al., 2009; Low et al., 2010). We believe prevention programs that teach emotion regulation and conflict resolution skills which have been linked to reductions in bullying (Beets et al., 2009; Frey et al., 2009; Li et al., 2011) could also help students repair and value peer relationships more, which according to our study, would also increase prosocial behaviors. These two variables, relational repair and relational value, were significant predictors of prosocial responding. Thus, our research suggests that teaching students emotion regulation and conflict resolution skills could go a long way to helping students repair relationships, which should lead to increased prosocial behavior and a likely reduction in retaliatory behaviors in response to rejection as found in prior research (Frey et al., 2015).

Another key element to all anti-bullying programs is the role of social support. This is also evidenced by the importance of a number of significant relationship variables such as relational value and relational repair, and sometimes alternative relationships as associated with increased prosocial responding. Students need to know that they can count on others for support and that the larger school climate along with peers, teachers, administrators can offer this support to them (Grapin et al., 2016). When social support is successfully implemented, it has likewise been found to increase prosocial behavior and decrease school safety concerns (Grapin et al., 2016). Finally, our study also highlights the importance of decreasing the influence of group affects and dynamics in schools as connected to retaliation for bullying as prior research has found (Gaertner et al., 2008; Frey et al., 2015). Addressing group dynamics in bullying would likely lead to reduced antisocial behavior and retaliatory behavior in response to aggression (Frey et al., 2015). To reduce intergroup aggression, an integration of both effective methods that reduce aggressive behavior and improve intergroup relations is needed (Hage et al., 2017; Palmer and Abbott, 2018). Some examples exist (Levy and Killen, 2010) including: changing social norms (Aboud and Joong, 2010; Perkins et al., 2011), getting students to recognize common superordinate group identity to counter segregated self-categorization (Gaertner et al., 2010), increasing intergroup contact to reduce negative attitudes (Griffin et al., 2012; Tauriac et al., 2013), modeling prosocial bystander interventions (Aboud and Joong, 2010), training youth to recognize multiple categorizations to combat dualistic us vs. them categorization (Cameron and Rutland, 2010), and affirming diversity and positive aspects of group identities to prevent out-group derogation (Wittig, 2010). Each of these approaches primarily addresses one contributing factor, not multiple factors. Thus, integrating these factors could provide a strong intervention (Aronson, 2000; Wernick et al., 2017). This could create a more positive social environment where students could begin to care for one another regardless of associated groups and their group membership. Finally, we wish to comment on the importance of reducing costs. We believe this is again connected to challenging present social norms that allow bullying to be acceptable in the first place. Second, it may be particularly important to ameliorate the associated costs such as loss in reputation or status for individuals who may already be at risk for isolation and self-harm.

### Conclusion

Our study makes a number of unique contributions (1) starting with being the first to test the full MMM, (2) plus conducting this study in two samples of diverse high school students, (3) who have experienced physical, verbal, relational, and/or cyber aggression, in addition to (4) examining the roles of groupness and (5) the outcome of self-harm. Our results suggest that there is high value to be placed on the importance of relationships and relationship skill-building when it comes to encouraging prosocial responding. Our study also highlights the importance of reducing the perception of costs associated with aggression, such as the loss in status, friendship, rank or “place” within a school. Anti-bullying prevention programs focused on social support could help to alleviate some of the perceived costs associated with aggressive rejection, including bullying. Reducing perceived costs could alleviate social pains youth experience, thereby reducing asocial and potentially self-injurious behavior. Finally, of importance to reducing antisocial behavior is reducing the likelihood that individuals perceive they are being targeted due to a social identity, have friends being co-victimized, or that others are targeting their peer groups. Prior research has found peers are likely to retaliate on the behalf of their friends (Frey et al., 2015), thus attending to and reducing group dynamics associated with aggression in schools could go a long way to reducing antisocial responses that ultimately contribute to cycles of aggression in schools.

## Data Availability Statement

The raw data supporting the conclusions of this article will be made available by the authors, without undue reservation.

## Ethics Statement

The studies involving human participants were reviewed and approved by Mississippi State University Institutional Review Board. Written informed consent to participate in this study was provided by the participants' legal guardian/next of kin.

## Author Contributions

MS-R and HS contributed to the overall study design of the paper. BP contributed to the analyses of the paper. JU contributed to the methods of data collection and initial cleaning of the data associated with the paper. HS led on the literature review. MS-R led on the discussion section of the paper. All authors contributed to the overall editing and final version of the manuscript.

## Conflict of Interest

The authors declare that the research was conducted in the absence of any commercial or financial relationships that could be construed as a potential conflict of interest.
