# Supplementary material for: When Does Rejection Trigger Aggression? A Test of the Multimotive Model
Source: Front Psychol. 2021 Jun 25;12:660973. doi: 10.3389/fpsyg.2021.660973 (PMC8267095; doi:10.3389/fpsyg.2021.660973)
Supplement: Supplementary file 1 [file Table_1.docx]

**Supplementary Tables**

| Supplementary Table 1. Items retained in SEM measurement model across datasets | | |
| --- | --- | --- |
| **Construal name**            Item text | Year 2 | Year 3 |
| **Affect/Self Esteem** |  |  |
| How much did this experience make you feel sad? | **Included** | **Included** |
| How much did this experience make you feel upset? | Excluded | **Included** |
| How much did this experience make you feel angry? | Excluded | **Included** |
| How much did this experience make you feel embarrassed? | **Included** | **Included** |
| How much did this experience make you feel afraid? | **Included** | Excluded |
| How much did this experience make you feel bad about yourself? | **Included** | **Included** |
| How much did this experience harm your self-esteem? | **Included** | **Included** |
| How much did this experience make you feel as though you have few good qualities? | Excluded | Excluded |
| **Cost of Rejection** |  |  |
| How much did this experience have a negative impact on you? | **Included** | **Included** |
| How much did this experience cost you anything? | **Included** | Excluded |
| How much did this experience cost you a loss physically (e.g., injury, illness)? | **Included** | Excluded |
| How much did this experience cost you a loss in reputation or status with friends/others? | **Included** | **Included** |
| How much did this experience cost you a loss in friendship? | **Included** | **Included** |
| How much did this experience cost you a loss in your personal belongings (e.g., possessions being damaged or stolen)? | **Included** | Excluded |
| How much did this experience cost you a loss in your performance (e.g., grades, success in sports/activities)? | **Included** | Excluded |
| **Alternative Relationships** |  |  |
| To what extent do you have other people to whom you can turn to? | **Included** | **Included** |
| To what extent do you have other people who you can count on? | **Included** | **Included** |
| To what extent do you have other people who will support you? | **Included** | **Included** |
| **Relationship Repairable** |  |  |
| To what extent do you have any interests in making the relationship you have with this person better? | **Included** | **Included** |
| To what extent do you feel you need to have a relationship with the person/persons who did this to you? | **Included** | **Included** |
| How much do you plan to repair the relationship with the person/persons who did this to you? | **Included** | **Included** |
| **Value of Relationships** |  |  |
| To what extent would you consider this person/persons a friend? | Excluded |  |
| How much would you consider this person/persons an enemy? (-) | Excluded |  |
| Because of this experience, it is more important than ever for me to maintain positive relationships. | **Included** | **Included** |
| Because of this experience, I would rather not have any close relationships. (-) | Excluded |  |
| Because of this experience, I have difficulty trusting others enough to form relationships. (-) | Excluded |  |
| Because of this experience, I find it difficult to value relationships (-) | Excluded |  |
| Because of this experience, it is important to me to find true friends | **Included** | **Included** |
| Because of this experience, I value the close relationships I have | **Included** | **Included** |
| **Chronicity/Pervasiveness** |  |  |
| I feel like this type of aggression happens to me all of the time. | **Included** | **Included** |
| I feel like these incidences are inescapable. | **Included** | **Included** |
| I feel like the aggression will continue no matter what I do. | **Included** | **Included** |
| I feel like this type of aggression rarely happens to me.  (-) | Excluded |  |
| I feel like these incidences can be easily avoided. (-) | Excluded |  |
| I feel like this type of aggression is not an issue for me. (-) | Excluded |  |
| **Rejection Unfairness** |  |  |
| Do you think the actions this person/persons took towards you were unfair? | **Included** | Excluded |
| Do you think the actions this person/persons took towards you were disrespectful? | **Included** | **Included*** |
| Do you think the actions this person/persons took towards you were mean? | **Included** | **Included*** |
| Do you think the actions this person/persons took towards you were unreasonable? | **Included** | Excluded |
| **Perceived Groupness** |  |  |
| To what extent was the person/persons who did this to you a member of the same group of people you hang out with? | Excluded | Excluded |
| To what extent was the person/persons who did this to you a member of a different group (people with whom you do not associate, peers you don’t hang out with)? |  | Excluded |
| To what extent was the person/persons who did this to you a member of any social group? | Excluded | Excluded |
| To what extent do you feel the person’s/persons’ actions were the typical behaviors of the group to which they belong? | Excluded | Excluded |
| How much did the group as a whole try to cause you harm? | Excluded | Excluded |
| How much do you feel the actions of the person who harmed you are similar to actions of others at the school? | Excluded | Excluded |
| To what extent do you feel you were targeted because you belong to a certain social group? | Excluded | Excluded |
| How typical is it for other members of your social group to be targeted by the same person(s) who harmed you? | **Included*** | **Included*** |
| How much does the harm done to your friends harm you? | **Included*** | Excluded |
| How much do you feel your social group experiences this type of aggression from others at school? | Excluded | **Included*** |
| **Prosocial Behavior** |  |  |
| Trying to help others deal with a similar problem. | Excluded | Excluded |
| Going to someone (e.g., parent, teacher, friend) for help. | Excluded | Excluded |
| Working things out with the person/persons who were aggressive towards me. | Excluded | Excluded |
| Forgiving the person(s) who hurt me. | Excluded | Excluded |
| Getting involved in new activities. | **Included** | **Included** |
| Trying to make new friends. | **Included** | **Included** |
| Turning to work or other activities to help you manage things. | **Included** | **Included** |
| **Withdrawal Behavior** |  |  |
| Trying to avoid situations where I have to be with other people. | **Included** | **Included** |
| Keeping to myself. | **Included** | **Included** |
| Thinking of ways to avoid seeing people. | **Included** | **Included** |
| Trying to “disappear.” | **Included** | **Included** |
| Staying away from the person(s)/group who was aggressive towards me. | Excluded | Excluded |
| **Antisocial Behavior** |  |  |
| Confronting the person/persons who hurt me. | Excluded | Excluded |
| Doing to others what was done to me. | Excluded | **Included** |
| Saying negative things about the person/persons to other people. | **Included** | **Included** |
| Figuring out a way to get back at them. | **Included** | **Included** |
| Saying mean things to the person(s) who hurt me. | **Included** | **Included** |
| **Self-Harm Behaviors** |  |  |
| Thinking about hurting myself. | **Included** | **Included** |
| Stop doing some activities I once enjoyed. | **Included** | **Included** |
| Injuring myself. | **Included** | **Included** |
| Engaging in behaviors that put my life at risk | **Included** | **Included** |
| Notes: blank spaces indicate that an item was not asked on a particular survey; *item loadings were restricted to be equal to keep all measures locally identified. | | |
