# Supplementary material for: When Does Rejection Trigger Aggression? A Test of the Multimotive Model
Source: Front Psychol. 2021 Jun 25;12:660973. doi: 10.3389/fpsyg.2021.660973 (PMC8267095; doi:10.3389/fpsyg.2021.660973)
Supplement: Supplementary file 2 [file Table_2.docx]

**Supplementary Tables**

| Supplementary Table 2. Standardized Structural Path Loadings from Planned Analyses, All Paths Estimated | | | | |
| --- | --- | --- | --- | --- |
|  |  | Predicted Direction | Year 2 | Year 3 |
| Prosocial Responses | Cost | + | .04 | .08 |
|  | Alternative Relationships | - | .18*** | .09 |
|  | Relationship Repairability | + | .20*** | .27*** |
|  | Value | + | .19*** | .27*** |
|  | Chronicity | 0 | .09 | -.01 |
|  | Unfairness | 0 | -.04 | .06 |
|  | Groupness | ± | .06 | .06 |
| Asocial Responses | Cost | 0 | .24*** | .44*** |
|  | Alternative Relationships | + | -.10 | -.02 |
|  | Relationship Repairability | - | -.04 | -.07 |
|  | Value | - | .21*** | .09 |
|  | Chronicity | + | .09 | .21** |
|  | Unfairness | 0 | -.07 | -.09 |
|  | Groupness | ± | .12 | -.02 |
| Antisocial Responses | Cost | 0 | .11 | .10 |
|  | Alternative Relationships | 0 | -.07 | -.05 |
|  | Relationship Repairability | - | .06 | .11 |
|  | Value | - | .05 | -.03 |
|  | Chronicity | 0 | -.01 | -.02 |
|  | Unfairness | + | .06 | .06 |
|  | Groupness | ± | .28*** | .30** |
| **p*<.05; ***p*<.01; ****p*<.001 | | | | |
